# Understanding barriers and facilitators to implementation of psychosocial care within orthopedic trauma centers: a qualitative study with multidisciplinary stakeholders from geographically diverse settings

**DOI:** 10.1186/s43058-021-00208-8

**Published:** 2021-09-15

**Authors:** Ana-Maria Vranceanu, Jafar Bakhshaie, Mira Reichman, James Doorley, Ryan A. Mace, Cale Jacobs, Mitchel Harris, Kristin R. Archer, David Ring, A. Rani Elwy

**Affiliations:** 1 Integrated Brain Health Clinical and Research Program, Department of Psychiatry, Massachusetts General Hospital, 1 Bowdoin Square, 1st Floor, Boston, MA USA; 2 Harvard Medical School, Boston, MA USA; 3 Department of Orthopaedic Surgery & Sports Medicine, College of Medicine, University of Kentucky, Lexington, KY USA; 4 Department of Orthopaedic Surgery, Massachusetts General Hospital, Boston, MA USA; 5 Department of Orthopaedic Surgery, Center for Musculoskeletal Research, Vanderbilt University Medical Center, Nashville, TN USA; 6 Department of Physical Medicine and Rehabilitation, Osher Center for Integrative Medicine, Vanderbilt University Medical Center, Nashville, TN USA; 7 Department of Surgery and Perioperative Care, Dell Medical School, The University of Texas at Austin, Austin, TX USA; 8 Department of Psychiatry and Human Behavior, Alpert Medical School, Brown University, Providence, RI USA; 9 Center for Healthcare Organization and Implementation Research, VA Bedford Healthcare System, Bedford, MA USA

**Keywords:** Orthopedic, Orthopaedic, Musculoskeletal, Traumatic injury, Surgeons, Medical provider, Focus groups, Qualitative

## Abstract

**Background:**

Psychosocial factors are pivotal in recovery after acute orthopedic traumatic injuries. Addressing psychosocial factors is an important opportunity for preventing persistent pain and disability. We aim to identify barriers and facilitators to the implementation of psychosocial care within outpatient orthopedic trauma settings using the Consolidated Framework for Implementation Research (CFIR) and Proctor’s taxonomy of implementation outcomes, and to provide implementation strategies derived from qualitative data and supplemented by the Expert Recommendations for Implementing Change.

**Methods:**

We conducted live video qualitative focus groups, exit interviews and individual interviews with stakeholders within 3 geographically diverse level 1 trauma settings (*N* = 79; 20 attendings, 28 residents, 10 nurses, 13 medical assistants, 5 physical therapists/social workers, and 3 fellows) at 3 trauma centers in Texas, Kentucky, and Massachusetts. We used directed and conventional content analyses to derive information on barriers, facilitators, and implementation strategies within 26 CFIR constructs nested within 3 relevant Proctor outcomes of acceptability, appropriateness, and feasibility.

**Results:**

Stakeholders noted that implementing psychosocial care within their practice can be acceptable, appropriate, and feasible. Many perceived integrated psychosocial care as crucial for preventing persistent pain and reducing provider burden, noting they lack the time and specialized training to address patients’ psychosocial needs. Providers suggested strategies for integrating psychosocial care within orthopedic settings, including obtaining buy-in from leadership, providing concise and data-driven education to providers, bypassing stigma, and flexibly adapting to fast-paced clinics.

**Conclusions:**

Results provide a blueprint for successful implementation of psychosocial care in orthopedic trauma settings, with important implications for prevention of persistent pain and disability.

**Supplementary Information:**

The online version contains supplementary material available at 10.1186/s43058-021-00208-8.

Contributions to the literature

- In this multi-site qualitative study of orthopedic trauma providers, we identified key strategies to facilitate implementation of psychosocial care into orthopedic trauma practices.
- We integrate the Consolidated Framework for Implementation Research with Proctor’s implementation outcomes as a novel approach to comprehensively characterize the barriers and facilitators among implementation determinants that could impact implementation outcomes.
- These data serve as a blueprint for maximizing successful implementation of psychosocial care and aligning orthopedic trauma practices with evidence-based biopsychosocial models of care.

## Background

Musculoskeletal traumatic injuries are a major public health problem [1]. The impact of traumatic injuries extends beyond immediate physical health, as approximately 20–50% of patients go on to develop persistent (chronic) pain and disability [2, 3], disproportionate to residual pathophysiology. Patients with greater pain and more functional limitations are likely to pursue additional surgeries and medical procedures with questionable potential for benefit, resulting in increased health care costs and a significant public health burden [4, 5].

Recovery after a traumatic injury is a complex process that extends beyond the severity of the physical injury itself. The biopsychosocial model [6] recognizes that biological, social, and psychological factors are interrelated and contribute together to the recovery process and long-term outcomes. Mounting evidence shows that misconceptions and distress (e.g., catastrophic thinking, fear of movement, depression, and posttraumatic stress) are important *modifiable* risk factors for persistent pain and functional limitations after traumatic musculoskeletal injuries, regardless of the injury severity [3, 7, 8], location [9, 10], and type [11, 12]. Recognizing these modifiable risk factors early creates an opportunity to intervene with patients who are at risk for persistent pain and disability in the acute post-injury phase, when psychosocial treatments are most effective [13, 14].

Despite the strong evidence for the role of psychosocial factors in recovery after traumatic injury, these factors are untreated or undertreated in most patients [15]. The Lower Extremity Assessment Project, a large prospective study of patients with orthopedic trauma, showed that while 50% endorsed psychological distress 3 months post-injury and 42% 2 years later, only 12% had received any mental health care early post-injury with numbers increasing only to 22% by the 2 year mark [15]. In 2019, the American Association of Orthopedic Surgery in partnership with the Major Extremity Trauma Research Consortium [16] developed clinical practice guidelines that recommended accounting for psychosocial factors when caring for people with traumatic injuries. Further, existing evidence supports the cost-effectiveness of the integration of psychosocial care within orthopedic trauma care at both societal and organizational levels [17–19]. However, implementation of these guidelines into orthopedic trauma settings has been severely limited [18].

Multiple factors known to prevent the successful implementation of evidence-based clinical practice guidelines in medical practices have been documented, including providers’ resistance, negative attitudes and lack of knowledge, skills, and organizational management support and resources [20]. Within the general surgical field, prior research has shown opposition to innovation from surgeons [21, 22]. Orthopedic surgeons may be particularly resistant to implementation of new clinical guidelines because they tend to prefer to retain substantial autonomy over their work practices and challenge external interventions [23]. In a survey of 350 orthopedic surgeons, 90% were “somewhat” or “very likely” to notice psychological factors, but only 60% were “somewhat” or “very likely” to refer their patients to psychological treatment [24]. Surgeons noted lack of time, mental health stigma, and feeling uncomfortable making referrals as barriers. Qualitative research among orthopedic trauma surgeons and staff is needed to gain a nuanced understanding of setting-specific barriers, facilitators, and implementation strategies to allow for the successful integration of psychosocial care in orthopedic trauma settings, consistent with AAOS guidelines.

The Consolidated Framework for Implementation Research (CFIR) [25] provides a framework for *identifying* and *reporting* on implementation determinants from the perspectives of stakeholders that would be impacted by incorporation of psychosocial care within orthopedic trauma settings. Additionally, Proctor’s taxonomy of implementation outcomes [26] provide a framework for *measuring* the success of implementation processes across multiple implementation domains including (1) *acceptability* (how tolerated psychosocial interventions would be within orthopedic trauma settings), (2) *appropriateness* (how relevant implementing psychosocial interventions would be within orthopedic trauma settings), and (3) *feasibility* (the extent to which psychosocial interventions could be successfully implemented within orthopedic trauma settings).

Integration of these two frameworks provides a novel approach to comprehensively characterize the barriers and facilitators among implementation determinants (CFIR) that could directly impact the specific implementation outcomes (Proctor) that would be used to determine the success of the implementation process, before engaging in concerted efforts toward implementation of a clinical innovation. For this particular study, we were interested in psychosocial care in general, rather than a specific treatment modality or care model (e.g., psychotherapy referrals versus care delivered within the orthopedic trauma setting, psychologist versus social worker delivered care) given prior survey data showing general challenges of orthopedic surgeons with psychosocial aspects of recovery.

We aimed to conduct a qualitative study at three geographically diverse outpatient orthopedic trauma centers to understand multidisciplinary stakeholders’ perceptions of barriers and facilitators to the implementation of psychosocial care. We also sought to identify potential implementation strategies to overcome barriers and capitalize on facilitators, using both our qualitative data and the Expert Recommendations for Implementing Change (ERIC) [27–29], a taxonomy of implementation strategies. Results will inform implementation of psychosocial care within orthopedic trauma settings to maximize outcomes for patients, surgeons, staff, and the larger health care system.

## Methods

### Setting

Sites A, B, and C (anonymized) are level I trauma centers in Austin, Texas; Lexington, Kentucky; and Boston, Massachusetts. Human subject oversight was provided by the Institutional Review Board of Site C. We followed the Consolidated Criteria for Reporting Qualitative Research [30] guidelines in study presentation (Additional File 1).

### Participants

Participants were outpatient orthopedic trauma providers across the three sites. Recruitment was facilitated through presentations to departments by “surgeon champions,” representing a purposive sampling approach. Orthopedic providers were eligible for study inclusion if they were directly involved in the care of outpatients with acute musculoskeletal injuries (e.g., fracture, dislocation, rupture) within any of the three level 1 trauma centers. Completion of an eligibility screening survey emailed to participants constituted implied consent for focus group participation.

The screening survey was distributed to 94 providers, of which 88 (94%) completed the survey and consented to participation. Of those consented, 79 (90%) participated in qualitative data collection (20 attending surgeons, 28 residents, 10 nurse practitioners/registered nurses/physician assistants, 13 medical assistants, 5 physical therapists/social workers, and 3 clinical research fellows). Nine providers (10%) consented but did not attend a focus groups due to planned or unexpected scheduling conflicts. Table 1 displays participant characteristics.

**Table 1:** Participant characteristics (*N* = 79)

Sex	Surgeons/residents (***N*** = 48)	Nurses/support staff (***N*** = 31)
Male	44 (91.7%)	10 (32.3%)
Female	3 (6.3%)	21 (67.7%)
Other	1 (2.1%)	0 (0%)
**Age**		
25–39	32 (66.7%)	20 (64.5%)
40–55	13 (27.1%)	10 (32.3%)
56–65	2 (4.2%)	1 (3.2%)
66–75	1 (2.1%)	0 (0%)
**Race**		
White/Caucasian	35 (72.9%)	22 (71.0%)
Black/African American	4 (8.3%)	3 (9.7%)
Asian/Asian American	6 (12.5%)	0 (0%)
Multi/other	3 (6.3%)	6 (19.4%)
**Ethnicity**		
Hispanic/Latino	1 (2.1%)	12 (38.7%)
Non-Hispanic/Latino	47 (97.9%)	19 (61.3%)
**Self-reported mental health training**		
Yes	24 (50%)	16 (51.6%)
No	24 (50%)	15 (48.4%)

### Procedure

We conducted 18 focus groups (7, 8, and 3 at sites A, B, and C, respectively) with 76 participants (42, 21, and 13 at each site). We combined providers of several roles (e.g., nurse practitioners with physician assistants) to create groups within the target range of 4 to 8 participants. Department chiefs participated in individual interviews (*N* = 3; 30 min). Focus groups (60 min) were facilitated by trained staff via Zoom and were followed by optional (10 min) exit interviews using “breakout rooms”.

Our semi-structured qualitative script (Table 2) was developed iteratively by a multidisciplinary team including psychologists, orthopedic surgeons, and an implementation science expert. The script was designed to generate data related to strategies to maximize the relevant implementation outcomes among those delineated by Proctor [26] and overcome inner and outer setting implementation challenges when integrating psychosocial care, as delineated by CFIR [25] (Table 3). For this study, we were specifically interested in the most widely used Proctor outcomes [26, 31–33], namely (1) acceptability (tolerability of psychosocial interventions in this setting), (2) appropriateness (relevance of psychosocial interventions in this setting), and (3) feasibility (viability of implementation in this setting).

**Table 2:** Semi-structured focus group script domains and questions

Domains	Questions
1. Clinical flow	How would you describe the “clinical flow” in the outpatient orthopedic trauma practice to someone unfamiliar? Is there any variability within this typical patient flow? How does the current clinical flow suit your work style and preferences?
2. Past experiences implementing clinical innovations	What do you think about implementing clinical innovations as part of clinical care for people who seek care in outpatient orthopedic trauma clinics? Can you recall any clinical innovations that were implemented that were successful or unsuccessful, particularly if you have an example from recent years?
3. Perceptions of barriers/facilitators to patient recovery	What do you consider a “good outcome” for your patients? What are some patient factors that might impede recovery in your patients? What factors help your patients recover well?
4. Perceptions of psychosocial needs of orthopedic patients	What comes to mind when you think of the terms “psychological, mental health, or behavioral concerns”? How often do you notice psychological, mental health, or behavioral problems in your patients? Do you formally assess or screen patients for psychological problems? What do you think about the role of these factors in the recovery trajectory of your patients?
5. Comfort addressing psychosocial factors in orthopedic trauma patients	How do you address mental or behavioral health problems that you notice in your patients? Do you ever refer or initiate the connection of patients to mental or behavioral health services? What mental and behavioral health resources are you aware of that are potentially available to your patients? What would be an ideal scenario for addressing mental health factors for your patients?
6. Barriers and facilitators to psychosocial care integration within orthopedic departments	How supportive are you of integrating psychosocial care within the orthopedic practice? What do you see as the most significant barriers to the integration of psychosocial care within orthopedic departments?
7. Individual exit interview (optional)	Is there anything that you would like to share relevant to the discussion from the focus group that you did not share in the focus group for any reason? How was your experience in the focus group today?

**Table 3:** Barriers, facilitators, and implementation strategies for the implementation of psychosocial care in orthopedic settings

CFIR domain/construct	Barrier or facilitator	Explanation of barrier/facilitator	Implementation strategies to improve implementation outcomes	Representative quotation
**Acceptability**				
Inner setting/culture	Barrier	Value of maximizing clinic efficiency above all else and lack of acceptability of any innovation that might disrupt clinical flow	Streamline referral process to minimize disruption to clinical flow; capitalize on existing wait time in clinical flow; Educate on how psychosocial care might reduce patient follow-up needs (e.g., post-surgery calls and visits); solicit feedback from providers regarding integration within clinic flow Tailor strategies* Conduct educational meetings* Create a learning collaborative*	“Medicine has turned into this, you know, turn and burn. You only get paid per click, you ‘gotta get him in and out… It’s just that our time constraints are narrowed down so much that it tends to fall down the list of priorities, right?” — Surgeon, site A
	Facilitator	Emphasis on values-based care; desire to maximize patients’ wellbeing	Capitalize on the desire by providing education about the positive impact of psychosocial interventions and building collaborative alliances Identify and prepare champions* Recruit, designate and train for leadership*	“We’re not money driven. Our goal is not to do more surgeries. We like to treat the patient as a whole.” — Medical Assistant, site A
Inner setting/implementation climate	Barrier	Resistance to innovation in clinic; low receptivity, and no expectation that use of the innovation will be rewarded, supported, or expected	Provide relevant incentives that are tailored for the specific type of stakeholders (e.g., evidence of treatment efficacy for surgeons or provision of support from leadership for other health professionals) Conduct local needs assessment* Alter incentive/allowance structures*	“For me it’s always difficult doing it. Change is always difficult. There’s no stimulus to do it unless you feel is a definite effect, so if it’s unlikely how much of an effect in this lot of work that is going to happen…” — Surgeon, site C
	Facilitator	Openness to innovation in clinic	Invest in and seek support from health professionals who are express openness Identify early adopters*	“I’m all for it. I’m big on improvement and, kind of, you know, evolving my practice. So, I’m looking forward to it.” — Surgeon, site B
Inner setting/access to information and knowledge	Barrier	Providers’ lack of knowledge of the importance of psychosocial factors in patient recovery	Provide data-driven and concise education/resources (electronic resources, videos, or in-person communication preferred) to highlight existing empirical evidence Develop educational materials* Distribute educational materials*	“And a lot of the times providers don’t really take mental health all that seriously if that makes sense. Like, sometimes they’re … ‘Oh he’s just crazy.’” — Medical Assistant, site A
	Barrier	Providers recognize they have a rudimentary knowledge of mental health; systemic education barrier	Provide data-driven and concise education/resources (electronic resources, videos, or in-person communication preferred) on managing with psychosocial factors Conduct educational meetings* Conduct ongoing training*	“Sometimes [patients] also have a psychiatrist and they will explain to me that they’ve been put on different medication and how they’re feeling, but that’s the extent of my conversation with them. You know, I have very rudimentary knowledge of psychiatry from medical school and that’s all I resort to.” — Surgeon, site C
	Barrier	Lack of acceptability of lengthy/time-consuming communications and trainings	Ensure communication is concise, to-the-point; Take advantage of captive time (e.g., grand rounds, scheduled meetings) Develop educational materials* Distribute educational materials*	“Medicine is very evidence-based, and—especially surgeons are—I think being concise, and to-the-point is very important. If it’s, you know, a very long email or a very long flyer, it can easily get thrown by the wayside, so being concise and data-driven I think are the biggest things.” — Resident, site A
Characteristics of individuals/knowledge and beliefs about intervention	Barrier	Personal negative bias against mental health factors (i.e., stigma)	Provide tailored psychoeducation according to the providers level of knowledge and source of bias Conduct educational meetings* Develop educational materials*	“I’ll admit this upfront. You know, 20 years of military service when you talk about mental health, psychological, even though I incorporate into my treatment plan, actually I have a strong, negative, unconscious bias towards it.” — Surgeon, site A
	Facilitator	Understanding of the emotional toll of traumatic injuries; empathy for patients’ psychological needs	Identify and develop early collaborations with stakeholders who show enthusiasm and could potentially serve as champions Assess for readiness and identify facilitators*	“You just happened to get a couple of people on the line tonight who were kind of in tune to some psychosocial aspects of patients…I’m only attuned to it really, quite frankly… I wasn’t very sensitive to the psychosocial aspects of being a fracture patient until I was in fact myself a fracture patient.” — Surgeon, site B
	Facilitator	Previous experience in psychology or good training in medical school and residency to see/treat the whole patient rather than the bone or injury	Consider starting from more advance stages of implementation and the potential for serving as champions. Stage implementation scale up* Identify and prepare champions*	“We are sensitive providers, you know. I think we think of the whole patient… I don’t think it happened in residency, dependent on mentor, but in Med school, you’ve got to think of the whole patient, you can’t just think of the bone. You meet that injury, you’ve got to think about everything, and I keep getting reminded. I had pretty good mentors in residency who reminded me of that too.” — Surgeon, site B
Characteristics of Individuals/self-efficacy	Barrier	Heterogeneity of providers' comfort level discussing mental health factors and perceived importance of mental health factors	Provide individualized education to providers regarding bringing up mental health concerns to patients; Take advantage of captive time (e.g., grand rounds, scheduled meetings) Conduct ongoing dynamic training/consultation* Model and simulate change* Shadow other experts*	“People are going to come at it with different levels of, you know, how much they think mental health measures, you know, are important in incorporating recovery …it’s not something that was traditionally part of people’s training, and so I think people will just come at it from different perspectives.” — Resident, site A
Implementation process/engaging/opinion leaders	Facilitator	Influence of leadership on perspectives of acceptability of innovations for providers	Engage formal leadership and opinion leaders to facilitate buy-in Involve executive boards* Obtain formal commitments* Inform local opinion leaders*	“I think if you have leadership within the orthopedic trauma department to say, “This is a priority. We want you guys to start implementing this into your patient visits,” … That’s probably path to success.” — Research Personnel, site B
**Appropriateness**				
Intervention characteristics/adaptability	Barrier	Heterogeneity in patients’ social/cultural contexts	Flexibly attune to patients’ social/cultural identities in treatment content using a culturally-informed approach (e.g., tailor examples of pleasurable activities, consider appropriateness of mindfulness for trauma-exposed patients) Capture and share local knowledge* Tailor strategies* Conduct local consensus discussions*	“If there’s homework in the therapy sessions, be mindful of what they are. So, an example can be, you know, “Go out and take a walk in your neighborhood” … and be aware of what the person’s environment is and know what would be an appropriate intervention, culturally, and then what’s going on with the patients.” — Physical Therapist, site A
	Barrier	Communication barrier with non-English-speaking populations (e.g., Spanish-speaking, Arabic-speaking)	Provide resources available in multiple languages, translation services available, and racially/ethnically diverse mental health service providers Conduct local needs assessment* Promote adaptability*	“If you’re going to work on serving patients who speak other languages or more diverse population, having materials translated is really important. I know from my experience, there are times where I’ve had to translate materials and sometimes that can be really challenging for the provider.” — Social Worker, site A
Intervention characteristics/evidence strength or quality	Barrier	Skepticism about priority/ relevancy of psychosocial interventions for orthopedic patients	Provide education to medical providers on evidence base for psychosocial interventions Conduct educational meetings* Inform local opinion leaders* Conduct educational outreach visits* Distribute educational materials*	“My role is to make sure everything is right in terms of classical medicine…while appreciate the patient perception, I should first make sure that everything is right …then reassure them that what I do [in terms of medical treatment] is right…I am not going to introduce them to a nonclassical medicine route.” — Surgeon, site C
	Barrier	Skepticism treating non-specific psychiatric disease (i.e., treating general emotional distress)	Ensure individualized services available; Provide education to medical providers on evidence base for transdiagnostic psychosocial interventions Conduct educational meetings* Inform local opinion leaders* Conduct educational outreach visits* Distribute educational materials*	“You must realize that that goes against every aspect of medical care that we’ve been trained to do… just everybody who might not be feeling great that day is far too vague than dealing with any specific problem… grouping them together just doesn’t make any sense at all… Just strikes me as completely insane.” — Surgeon, site C
	Facilitator	Data-driven value of providers coupled with provider interest in patient functional outcomes	Capitalize on providers data-driven values by providing direct evidence on improvement in outcome following psychosocial interventions Develop academic partnerships*	“We’re in the age of evidence-based medicine, and if you have evidence to prove it that would work. … Orthopedic trauma itself is a very vast field with so many different personalities and characteristics, but no one can refute evidence.” — Surgeon, Site B
Intervention characteristics/complexity	Barrier	Concern for appropriateness of intervention for persons with low levels of education/literacy	Use simplified language or “lay” language; Incorporate figures and illustrations into educational materials Capture and share local knowledge* Develop a formal implementation blueprint* Model and simulate change*	“But you can’t—you can’t use big words—you can’t—I mean, you laugh—but, it’s—it’s the truth, like, you’re going to lose people—you can’t use big words… you can’t forget your population.” — Resident, site B
Outer setting/patient needs and resources	Barrier	Patients who do not have basic needs met (e.g., are experiencing homelessness, substance use, do not have access to food/safe space/transportation) or may not have ability/willingness to engage in psychosocial services	Enable flexibility in treatment pacing, duration, and content based on individualized needs to build rapport and “meet patients where they’re at” Conduct local needs assessment* Involve patients/consumers and family members* Obtain and use patients/consumers and family feedback*	“I think one thing I noticed on my psych rotation is that a lot of these folks are living a very teetering life where one unfortunate circumstance can have their life spin out of balance …. So, getting appropriate resources for them is really important.” — Resident, site B
	Barrier	Orthopedic team’s not prioritizing addressing psychosocial care within orthopedic care	Seek support from leadership for system change through provision of incentives and educational opportunities Assess for readiness and identify barriers and facilitators* Use advisory boards and workgroups*	“Our healthcare system is so fragmented, so I think, as a specialty practice, we don’t do as good of a job at addressing those needs… I think we have this view—it’s like, ‘Well, we’re orthopedics, we’re just treating that fracture, or that injury,’ and if the patient does have psych needs, it’s, you know, often kind of a culture as ‘Well, that’s for the PCP to, sort of, deal with, or that’s for the psychologist, or the psychiatrist.’” — Nurse, site C
	Barrier	Difficulty of determining which patients would benefit from integrated psychosocial care versus outside specialty providers	Exploring the specific characteristics of the setting and patient population to develop system of decision making, and referral tailored for individual patient. Conduct local needs assessment* Involve patients/consumers and family members*	“We see an ever-increasing number of patients. So, that brings up an interesting point—how do you refer people with psychological needs outside of the system and maintain some sort of working relationship? And then, how do you figure out which patients are going to benefit from staying within an interdisciplinary system versus getting their needs met from an outside referral?” — Physical Therapist, site A
	Barrier	Perception of lack of clear pathways to getting appointments to patients who express a mental health need	Collaborative clarification of the available resources and road map for referrals Conduct local consensus discussions*	“To just have the name if we needed somebody, like a local person to send people to… that might be helpful. I just feel like we have little partnerships with doctors around [the area] but we don’t really have a psychiatrist or anything like that already.” — Medical Assistant, site A
	Barrier	Lack of knowledge on how to engage patients to follow with outpatient services and goals (e.g., physical therapy, meds)	Enable treatment strategies to improve patients’ insight and motivation for engagement with care related practices. Prepare patients/consumers to be active participants* Intervene with patients to enhance uptake and adherence*	“We talk about non-compliance… So, it may seem really easy to say, ‘Take your meds, exercise, do these exercises and practice mindfulness,’ you know, that sounds like those are very smart goals and you can do those, but really understanding what their situation is and just being really intentional of how to help.” — Physical Therapist, site A
	Facilitator	Perception that psychosocial needs in patients are vast and under addressed	Consider as an avenue for developing constructive collaboration with the providers to address these needs Build a coalition*	“These people aren’t hiding, like, they are in plain sight. You see them in the trauma clinic, and you’re like ‘That is someone who is not coping well.’ … These are people who are struggling. I think they want help.” — Resident, site A
Outer setting/external policy and incentives	Facilitator	Telehealth as increasing accessibility of care	Capitalize on the increased accessibility to further disseminate the psychosocial interventions Change structure and equipment*	“I think prior to COVID I was like ‘Oh I don’t know about the videos’ but now, ever since COVID started and we had to do a lot more Zoom, I feel like patients are liking like these videos.” — Medical Assistant, site A
Inner setting/structural characteristics	Barrier	High patient volume and fast-paced clinic flow make implementation of innovations difficult	Streamline process for providers referring patients to psychosocial care; Solicit feedback from providers regarding integration within clinic flow Assess for readiness and identify barriers and facilitators* Change structure and equipment* Conduct cyclical small tests of change*	“You’ve tapped a trauma surgeon that is very busy on the bell curve, they’re top five percent as far as volume…You got to understand what goes along with that in a private practice and make sure that the metrics you’re looking for are well-defined, and you don’t vacillate from it.” — Surgeon, site A
	Facilitator	Multidisciplinary nature of department (e.g., embedded physical therapists, dieticians etc.); interest in being a “one stop shop”	Use as an opportunity to promote multidisciplinary collaboration Identify early adopters* Promote network weaving* Build a coalition*	“Another thing that’s different about [our site] is we not only have a primary surgeon, but we have our NPs and PAs. We also have a physical therapist, we have dietitians and social work, so there can be one patient who’s there for four hours, but they meet the surgeon, they meet the dietitian … We try to do like a one stop shop here for the patient so they can get everything as much as possible in one visit.” — Medical Assistant, site A
Inner setting/compatibility	Barrier	Concern regarding interference with surgeons’ clinical flow and workflow	Develop adaptive ways for integration of psychosocial referrals into the surgeons’ workflow by soliciting feedback from the providers Promote adaptability* Conduct local consensus discussions* Purposely reexamine the implementation*	“I think that success is going to come from being as non-obtrusive in your implementation as possible.” — Resident, site B
	Facilitator	Perceived relevance of psychosocial care for patients’ needs and acknowledgment that psychosocial care would reduce burden on surgeons to have mental health-related conversations with patients	Capitalize on this insight and seek avenues to facilitate implementation through building fruitful collaborations, preparing champions, etc. Assess for readiness and identify facilitators* Identify and prepare champions* Facilitation*	“In a perfect world, it would be wonderful if we had a psych team that was designated just for, like, trauma, that we could call, and they could see the patient and, you know, they’ve specialized in patients who have the mental health history, and then, on top of that, now they’re experiencing a traumatic injury, and, just, too, for the person who doesn’t have the psych history and experiences a trauma….I know that would generally benefit our providers, our patients, their outcomes, patient satisfaction—all of it—provider satisfaction, it would just be huge.” — Nurse, site C
	Facilitator	Providers’ desire for additional support in encouraging patients to develop resilience to pain and engage in activity despite pain	Ensure message of psychosocial services align with recovery messages of clinic providers through communication/education Build a coalition*	“I think the huge thing was safely getting involved in activity despite pain. Patients are afraid. A lot of times, if something hurts, they think that they’re, you know, damaging themselves, or they’re going to re-break something, or mess up their fixation, but I think the biggest thing is getting these patients up and out of bed, and being able to mobilize them, and any kind of, fight through to prevent them having contractures, or, you know, continued pain. I think that’s huge.” — Resident, site B
**Feasibility**				
Intervention characteristics/cost	Barrier	Patients’ insurance might have limited coverage for psychosocial care	Seek potential avenues to reduce cost in collaboration with local and regional stakeholders Alter patient/consumer fees* Access new funding* Use other payment schemes*	“I was going to say cost [as a barrier], and what their insurance covers.” — Resident, site A
	Barrier	Perception of need for large amount of funding by organization to implement any type of psychosocial care	Show data on how psychosocial care might be cost saving for organization Develop resource sharing agreements* Fund and contract for clinical innovation* Place innovation on fee for service lists/formularies* Involve executive boards*	“I’d be pretty open to it, but, again, it comes to funding. Like, who’s going to pay for the psychologist? You know, if this is an ortho-trauma provider, you know, that’s what our boss is going to look at, you know, they’re going to look at the bottom—bottom dollar, you know.” — Surgeon, site B
Inner setting/networks and communication	Barrier	Challenge to maintain communication regarding patient needs among multidisciplinary providers	Frequent reminders Organize clinician implementation team meetings* Promote network weaving*	“I mean, so this is a problem in being in an interdisciplinary environment, is there are so many people considering different pieces of one puzzle, and then we’re kind of relying on our interoffice communication to put it together.” — Physical Therapist, site A
	Facilitator	Use of screeners that can funnel patients into appropriate services in conjunction with orthopedic care after visits	Enable centralized center-wide screening methods for early identification of the need for psychosocial care Centralize technical assistance*	“We do have screening that occurs from a PROs perspective, before they come in… I’m thinking of a particular patient that I have—who, 2-3 visits in, it was clear to me that I was not going to be able to address this on my own, and I asked if she would be interested in talking to social work, and so she agreed and so then I requested social work involvement.” — Physical Therapist, site A
	Facilitator	Trust between doctors and clinical staff; Horizontal staff structure in which staff are encouraged to communicate observations to higher-ups	Encourage and develop a system of knowledge sharing and communication in the service of patients’ needs Capture and share local knowledge* Create a learning collaborative*	“We’re definitely comfortable speaking with each other and especially about patients. It’s a high priority to us… like I said, we do those questionnaires for anxiety and depression, you know, even if those look normal and I just get a weird vibe with maybe a patient mentioned something concerning, I have no problem bringing it up to the provider and saying ‘Hey, you might want to ask them about this because they said something about this.’” — Medical Assistant, site A
Inner setting/available resources	Barrier	Perception of lack of human resources to support integration of psychosocial care	Ensure adequate staff to facilitate referral process for psychosocial care and provision of psychosocial care; educate and collaborate with all types of providers in orthopedic department; ensure clear division of responsibilities Fund and contract for clinical innovation* Develop resource sharing agreements*	“Adding another job responsibility on to the trauma clinics—maybe some of the other clinics—but in the trauma clinic specifically it’s extremely hard already trying to do the job at hand.” — Medical Assistant, site A
	Barrier	Perceived lack of time in clinic flow to implement innovations (e.g., time for referral process or time to have “heart to heart” with patients)	Streamline process for providers referring patients to psychosocial care; Solicit feedback from providers regarding integration within clinic flow Capture and share local knowledge* Change structure* Develop resource sharing agreements*	“They would probably do well with like a 15-minute, you know, kind of heart-to-heart with the doctor. But when we see 35 people, you don’t have the time to do that with every single person…So, part of the problem is you just don’t have the time to make them feel better, which sounds really insensitive.” — Medial Assistant, site A
Inner setting/tension for change	Barrier	Despite the belief that psychosocial care is needed for orthopedic patients, the current situation does not seem as intolerable to the stakeholders	Provide evidence regarding the short term and long-term costs of delaying the implementation of psychosocial care model Facilitate relay of clinical data to providers* Inform local opinion leaders*	“How could we consider the addition of a new tool or something, even though we all acknowledge that it’s really a big deal, but we haven’t been able to break the inertia that it takes to incorporate certain things.” — Surgeon, site A
	Facilitator	Perceived urgency to address psychosocial needs in patients	Use these opportunities to capitalize on providers need and promote/move forward with the implementation of psychosocial care model Identify and prepare champions*	“Particularly in the pandemic, the needs are higher, and so I’ve heard from social workers that it’s really heavy and difficult to hear the trauma stories, process, problem solve—it feels a little bit heavier than before because of the difficulties of the pandemic.” — Social Worker, site A
Implementation process/engaging	Barrier	Individual nature of buy-in by providers (i.e., difficult to engage *all* providers)	Education should be engaging and motivational to increase chance of buy-in; engage formal leadership and opinion leaders to facilitate buy-in; build rapport/relationships with providers Make training dynamic* Facilitate relay of clinical data to providers* Inform local opinion leaders*	“Identify which surgeons on the trauma service want to participate…and then—and—and then—I don’t know if you could try to focus on those clinics—but that might be the best way to go.” — Nurse, site C
	Barrier	Stigma associated with mental health may impede patient uptake of services	Have doctors refer patients to services; Emphasize importance of psychosocial services for pain, recovery, and overall health; Hire providers who sound/talk like patients; Ensure patient privacy/confidentiality related to mental health discussions (i.e., do not introduce in front of family members) Intervene with patients to enhance uptake and adherence* Involve patients* Identify and prepare champions* Tailor strategies*	“We definitely have long conversations over avoiding the word ‘depression,’ you know, using the word like ‘feeling blue’ or ‘sad.’ I think people, when they see the words ‘anxiety’ and ‘depression,’ ‘mental health,’ they get scared and they think ‘Oh that’s, you’re getting too private now.’” — Medical Assistant, site A
	Barrier	Patients may have negative reaction to hearing psychosocial factors are a contributor to their pain; Patients may get message that pain is “all in their head”	Emphasize importance of psychosocial services for pain, recovery, and overall health with a focus on a mind-body framework Intervene with patients to enhance uptake and adherence* Involve patients and family members*	“Well, you know, we still live in a world where, you know, unfortunately, psychosocial issues are still considered taboo, and so I would say that when you start to say anything about treatments that involve anything related to the mental capacity, mental space, they assume you mean that you think they’re crazy and they’re not going to get—you know, that it’s all in their head.” — Nurse Practitioner, site A
	Barrier	Fast-paced nature of clinic makes it challenging for patients to open up about thoughts and feelings	Give psychosocial factors attention deserved by “owning” time during clinic flow when talking to patients Change structure and equipment* Promote adaptability*	“I think making sure to like, slow the process down, maybe, however you implement yourself into the process, because I think if you rush patients, they’re probably not going to be as open.” — Research Personnel, site B
	Facilitator	Patient interest in pain and doing anything to alleviate pain	Emphasize/educate patients that psychosocial factors are a primary contributor to pain Intervene with patients to enhance uptake and adherence* Obtain and use patients and family feedback* Prepare patients to be active participants*	“All of my patients care about their pain and their pain control, and honestly, when can they get their next pain medication refill, and so anything that, up front says that we have proven that, you know, these techniques are going to help, you know… ‘We believe that this is going to help your pain,’ honestly, that’s the big selling point that my patients need to hear.” — Nurse Practitioner, site A
	Facilitator	Buy-in from providers can help convince patients that psychosocial care is important	Engage formal leadership and opinion leaders to facilitate buy-in; have doctors refer patients to services Conduct educational meetings* Inform local opinion leaders* Make training dynamic*	“And so, I think having—having the surgeon—kind of, providing them with the tools to at least, you know, bring up the topic and endorse the problem itself, directly and deliberately, will be an important part of patient enrollment and compliance.” — Resident, site A
	Facilitator	Residents are available who are more malleable than attendings	Enable engaging scientific discussions to highlight the need for psychosocial care Conduct educational outreach visits*	“So, I mean, I think you guys are doing a good thing and trying to target academic institutions, especially where residents are involved, because if you can change behavior in residence, then that’ll be a big impact.” — Research Personnel, site B

Implementation strategies denoted with an asterisk are derived from ERIC as opposed to directly from qualitative data. Representative quotations are provided for each barrier and facilitator, identified by participant role and site

Focus groups were facilitated by predoctoral and postdoctoral research fellows in psychology with training by the multidisciplinary team and no prior relationship with participants (AMV, JB, JD, RAM). Focus groups, exit interviews, and individual interviews were audio recorded and transcribed verbatim by research assistants.

### Data analysis

Our data analysis involved two types of content analysis: directed, to identify implementation determinants and outcomes, and conventional, to identify implementation strategies [34]. For the directed content analysis approach, we developed a coding framework by combining all 39 CFIR implementation determinants and the three Proctor implementation outcomes (acceptability, appropriateness, feasibility), thereby selecting codes a priori based on these conceptual frameworks. Given the different orientations of these two frameworks, with CFIR focusing on determinants and Proctor framework focusing on the success of implementation strategies, we decided to integrate both frameworks in order to achieve a more comprehensive understanding of different aspects of psychosocial care integration in orthopedic settings. We believe this expanded theoretical coverage of barriers and facilitators as related to their determinants (CFIR constructs) as well as their respective implementation outcomes (Proctor constructs) will provide for more efficient implementation planning [35]. We also allowed for new codes to emerge during the coding process, but no new codes emerged within the scope of our research questions. Using NVivo software as a data management tool, three coders systematically applied the coding framework to transcripts. Each transcript was independently coded by two coders. Coders met to discuss discrepancies in coding and reach resolution. Coder agreement was excellent (Kappa = 0.93).

We took a collaborative approach to data interpretation. Four team members (JB, JD, MR, RAM) looked at the charted data within each code (CFIR determinants and Proctor outcomes) and identified emerging barriers and facilitators to implementation. We aimed to comprehensively capture all barriers and facilitators that emerged, without concern for the frequency with which barriers and facilitators were raised. We then sought to align barriers and facilitators with specific implementation determinants *and* outcomes. When consensus was not reached as to which framework constructs aligned with identified barriers or facilitators, the coding team engaged in discussion to arrive at a consensus-based, collaborative decision to categorize them under the constructs that were most relevant, after consulting the definitions of these dimensions and with considerations for all options [34]. At times, we also observed multifaceted barriers [36] related to different aspects of each framework, and in these cases, we coded the identified barrier to multiple constructs of each relevant framework. Similarly, we intentionally allowed some barriers and facilitators to contradict and to represent the breadth and diversity of opinions expressed by participants. For the identification and selection of implementation strategies, we used conventional content analysis, allowing descriptions of implementation strategies to emerge from participants’ own words regarding how to overcome barriers and capitalize on facilitators. We supplemented implementation strategies extracted from the qualitative data with implementation strategies selected from ERIC using the CFIR-ERIC matching tool [37], to ensure a comprehensive, data-driven approach to implementation strategy identification and selection.

## Results

**Fig. 1:**
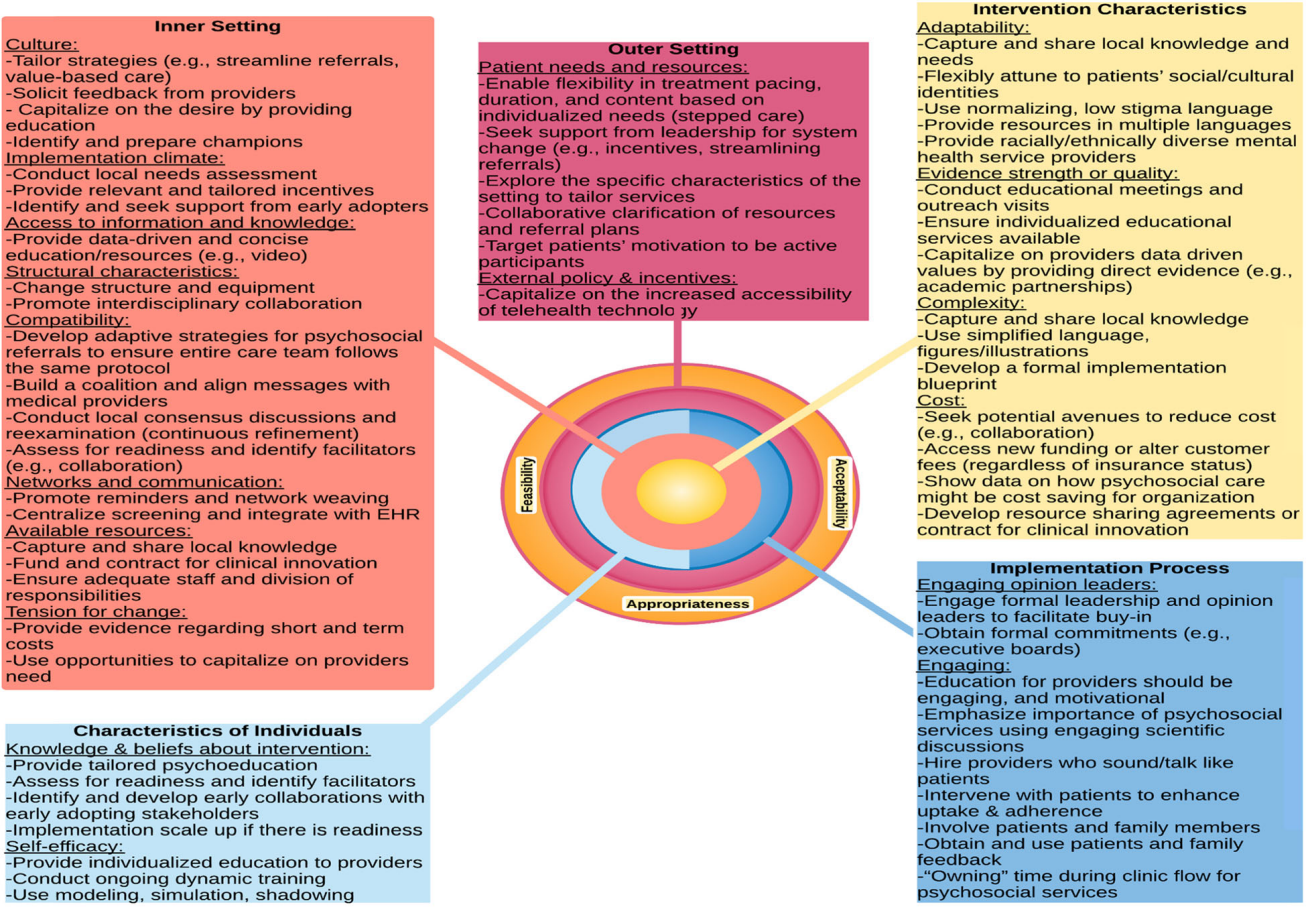
Recommendations for implementation of psychosocial care within orthopedic trauma within CFIR constructs and domains

Data best fit within 26 of the 39 CFIR constructs. The remaining 13 constructs either did not have any pertinent data or they had little data that also fit within one of the 26 constructs. These 26 constructs most pertinent to implementation of psychosocial care within orthopedic settings span all 5 CFIR domains. We also identified determinants corresponding to each of the three Proctor implementation outcomes (acceptability, appropriateness, feasibility) in our coding framework (Table 3; Fig. 1). Below, we discuss implementation determinants according to the CFIR constructs (italicized in paragraph) they best represent, nested within Proctor implementation outcomes (section headings). We also present key implementation strategies to address these determinants derived directly from the qualitative data. Table 3 also presents additional more general implementation strategies identified from ERIC.

### Acceptability

Regarding *culture*, many participants highlighted value-based approaches to care that focused on patients’ well-being, including mental health, over efficiency, or financial profit. On the other hand, some noted that the culture in their clinics values efficiency and that any innovation that may decrease efficiency would not be viewed favorably. Participants proposed educating providers on the importance of psychosocial care as well as ensuring a streamlined referral process. In terms of *implementation climate,* most participants conveyed openness to clinical innovations broadly and psychosocial interventions specifically. However, some expressed resistance to clinical innovations. Regarding *access to information and knowledge*, participants voiced concerns about orthopedic providers’ lack of knowledge about the importance of psychosocial factors in patient care and the belief that a systemic barrier in medical education contributes to this knowledge gap. As one surgeon described, “You know, I have very rudimentary knowledge of psychiatry from medical school and that’s all I resort to” (site C). Participants proposed providing providers with concise, data-driven psychoeducational resources in virtual formats.

With respect to *knowledge and beliefs about psychosocial interventions*, participants noted that orthopedic providers tend to understand the psychological toll of traumatic injuries. Some reported previous experience with psychology, while a few reported general bias against the relevance of mental health factors. In terms of *self-efficacy*, participants noted the wide range of variability among orthopedic providers regarding ability to discuss psychosocial factors, suggesting that psychosocial interventions may be more easily integrated into some surgeons’ clinics than others. Regarding *engaging* of *opinion leaders*, participants highlighted that buy-in from leadership within the orthopedics department is key for engaging the rest of the providers in supporting psychosocial care implementation. One research staff member described, “If you have leadership within the orthopedic trauma department to say, ‘This is a priority. We want you guys to start implementing this into your patient visits’ … That’s probably the path to success” (site B).

### Appropriateness

Participants expressed concerns about the *adaptability* of psychosocial interventions to racially/ethnically diverse patients, including non-English speakers. They suggested that psychosocial care should be tailored to patients’ sociocultural identities, provided in multiple languages, with on-site translation and racially/ethnically diverse clinicians. Regarding *evidence strength or quality*, several participants presented skepticism regarding the relevance of addressing psychosocial factors to improve patient outcomes given the abundance of effective medical options. However, many participants noted that surgeons and other providers are highly receptive to empirically supported interventions to improve patient functional outcomes. They suggested highlighting the evidence base for psychosocial interventions to generate provider buy-in. As one surgeon noted, “We’re in the age of evidence-based medicine… no one can refute evidence” (site B). In terms of *complexity*, some participants expressed concerns about whether psychosocial interventions are appropriate for patient with lower levels of education and health literacy and encouraged the use of “lay language” with illustrations.

Related to *patient needs and resources,* some participants noted skepticism that participants would follow-up with outpatient mental health care (e.g., due to transportation barriers or homelessness). To circumvent these barriers, participants suggested flexibly adapting psychosocial treatment pacing and duration to patients’ circumstances and prioritizing psychoeducation to enhance motivation and adherence. As one resident described, “I think one thing I noticed on my psych rotation is that a lot of these folks are living a very teetering life where one unfortunate circumstance can have their life spin out of balance … So, getting appropriate resources for them is really important*.*” (site B). Related to *external policy and incentives*, participants mentioned that increased uptake of telehealth practices due to COVID-19 can facilitate access to psychosocial care and enhance integration.

Regarding *structural characteristics,* participants noted that fast-paced clinic flow and high patient volume challenge the integration of psychosocial care into orthopedic trauma clinics. They suggested streamlining the referral process and soliciting feedback throughout the implementation process. Some reported that the multidisciplinary nature of their clinics might facilitate integration, as patients and providers already view their clinic as a “one-stop shop” for multiple forms of care (e.g., physical therapy, dietetics). Regarding *compatibility*, providers perceived a need for additional support to help patients develop healthy pain coping strategies. They acknowledged that integrated psychosocial care would reduce this burden on orthopedic providers.

### Feasibility

Participants expressed concerns about the *cost* of psychosocial care integration. Some noted that patients’ insurance might not cover psychosocial care and departmental funds may be required to make psychosocial intervention part of standard care. They suggested presenting data to departmental and organizational leadership demonstrating the cost-effectiveness of psychosocial care integration. Regarding organizational *networks and communication*, some participants expressed concerns about effectively communicating patients’ psychosocial needs within their large multidisciplinary team and suggested integrating psychosocial care information within electronic health records. Some participants mentioned that their clinics do have existing channels for communication about patient needs across providers, which would facilitate communication from medical staff to surgeons regarding patient psychosocial concerns.

In terms of *available resources*, participants expressed concerns about insufficient time and human resources to feasibly integrate psychosocial care. As one medical assistant shared, “Adding another job responsibility on to the trauma clinic… in the trauma clinic specifically it’s extremely hard already trying to do the job at hand” (site A). To circumvent these barriers, they highlighted the importance of ensuring a clear division of responsibilities and ensuring adequate staff to facilitate referrals. Relevant to *tension for change*, participants expressed differing opinions regarding the urgency of addressing psychosocial needs in orthopedic patients. Some expressed an urgent need while others noted that the current situation does not seem intolerable enough to require innovation.

Participants reported barriers to the process of *engaging* orthopedic providers in the process of integrating psychosocial care, including heterogeneity of provider preferences for psychoeducational materials. Participants suggested engaging departmental chiefs and opinion leaders as a key strategy for increasing buy-in. Relevant to *engaging* patients in psychosocial care, participants raised concerns regarding mental health stigma and limited willingness of patients to “open up” in the context of fast-paced orthopedic visits. As one medical assistant described, “I think people, when they see the words ‘anxiety’ and ‘depression,’ ‘mental health,’ they get scared and they think ‘Oh that’s, you’re getting too private now’” (site A). Strategies to circumvent these barriers included hiring psychosocial care providers who share racial and cultural identities with patients and emphasizing the importance of psychosocial care for pain and recovery to patients.

## Discussion

We conducted a qualitative study with orthopedic trauma providers at 3 geographically diverse level 1 outpatient trauma clinics to understand barriers and facilitators to integrating psychosocial care within usual outpatient orthopedic trauma care and identify implementation strategies to overcome barriers and capitalize on facilitators from the perspectives of stakeholders as well as from ERIC [29]. By providing information on CFIR determinants organized by Proctor implementation outcomes, we demonstrate the value of integrating these two frameworks for the analysis of qualitative data, to provide a more complete picture of the challenges to implementing psychosocial care within orthopedic settings. We observed high enthusiasm for this qualitative study within participating orthopedic trauma departments; 94% of individuals approached consented and 90% of these individuals participated in the focus groups.

Overall, providers appreciated the role of psychosocial factors in recovery after orthopedic trauma and noted that implementing psychosocial care within their practice can be acceptable, appropriate, and feasible. To be acceptable, psychosocial screening and treatment must be seamlessly integrated within the fast-paced clinic flow, with clear delineation of each provider’s role. Because readiness for implementation is heterogenous, it is important to provide tailored education (e.g., brief videos or presentations) on the process and scientific evidence for psychosocial care to surgeons and staff, in addition to patients. Early adopters can serve as “champions” for these efforts — catalyzing cultural change and correcting any negative biases. Providers with greater self-efficacy regarding psychosocial care could lead trainings and offer shadowing experiences. Early and sustained support from leadership is key.

Results suggest that to be appropriate, interventions must be tailored for content and delivery (e.g., lay language that normalizes challenges to decrease mental health stigma). Stepped care models have been successful in other settings [38] and may provide useful in triaging patients to appropriate levels of care including outside referrals for those with complex psychosocial needs (e.g., homelessness, severe psychopathology, substance use). Because orthopedic providers may not know about evidence-based psychosocial treatments, it is important to provide brief education including how they differ from the typical surgical protocol. For example, psychological treatments have largely moved away from treating one discrete condition (e.g., depression) toward process-based psychosocial interventions [39, 40]. These interventions target core constructs (e.g., pain catastrophizing) that cut across a variety of medical (types of orthopedic trauma injuries) and psychological (depression, anxiety, and posttraumatic stress) conditions. Although empirical evidence for the role of these interventions for orthopedic trauma is emerging [41, 42], these approaches may be counterintuitive to surgeons trained to perform specific surgeries (e.g., extramedullary fixation device) for specific diagnoses (e.g., hip fracture). Refining protocols for psychosocial intervention implementation over time based on lessons learned and flexibly tailoring them to the resources already available in each clinic will help circumvent barriers related to heterogeneity of provider buy-in and resource availability.

Results show that increasing feasibility of psychosocial care for orthopedic trauma patients will require ensuring that psychosocial treatment is provided regardless of patients’ insurance status. When possible, efforts should be made to reduce costs, access new funding sources, or develop resource sharing agreements to reduce patient fees. Indeed, early psychosocial care can decrease long-term healthcare costs for orthopedic patients. Educating leadership on the long-term cost-effectiveness of psychosocial care in terms of both reducing medical care utilization over time and reducing surgeon burden, while also ensuring a streamlined process with enough staff support is important. The use of telehealth can increase accessibility.

The current study has several strengths and limitations. First, we conducted the largest qualitative study on this topic to our knowledge. Second, we captured diverse experiences by including diverse stakeholders across 3 level 1 trauma settings. Third, we used evidence-based implementation frameworks and combined CFIR with Proctor and ERIC to more thoroughly understand barriers and facilitators to implementation, yield as many implementation strategies as possible across levels of the organizations (e.g., individual providers, clinic culture, patient needs and resources), and organize implementation strategies to guide future work. Notably, we also derived implementation strategies directly from our qualitative data, which were generally consistent with the strategies suggested by ERIC, increasing confidence in our findings. A challenge that we encountered in the analysis was how best to make decisions about where barriers and facilitators identified from the data fitted best, particularly when they could be mapped onto more than 1 CFIR construct. In such cases, we mapped information onto the construct that we considered to be the best ‘fit’. Our sample was primarily young, White and Non-Hispanic/Latino, which could impact the transferability of our findings to older and non-White populations. The percent of women surgeon participants was also low, although we enrolled all available women surgeons. Future qualitative studies should aim to use more diverse samples. Lastly, while our goal was to explore 3 of the Proctor outcomes (acceptability, appropriateness, and feasibility), future studies should also qualitatively explore information on outcomes of cost and sustainability. Toward this end, qualitative interviews with administrative leaders and external stakeholders (e.g., payers) would provide valuable, in-depth information to further maximize overall success of efforts toward implementation of psychosocial care within orthopedic trauma settings.

## Conclusions

We found widespread support for psychosocial care integration within orthopedic trauma settings. Multidisciplinary providers perceived psychosocial care as crucial for optimizing patient outcomes and reducing provider burden, noting they lack the time and specialized training to fully address patients’ psychosocial needs. Providers also perceived that psychosocial care integration would be challenging due to fast-paced clinical flow. By integrating CFIR, Proctor, and ERIC frameworks, we identified actionable strategies for integrating psychosocial care, including obtaining buy-in from department leadership, succinctly communicating the importance of psychosocial care to providers, tailoring interventions to patients from diverse backgrounds, bypassing stigma, and flexibly adapting to fast-paced clinical flow. Mental health practitioners, clinical researchers, and implementation scientists can use these data as a blueprint for maximizing successful implementation of psychosocial care and aligning orthopedic trauma practices with evidence-based biopsychosocial models of care.

## Supplementary Information

**Additional file 1.** COREQ (COnsolidated criteria for REporting Qualitative research) Checklist

## Data Availability

The datasets used and/or analyzed during the current study are available from the corresponding author on reasonable request.

## Abbreviations

CFIR: Consolidated Framework for Implementation Research
ERIC: Expert Recommendations for Implementing Change
